# Identification of specific B cell linear epitopes of mycoplasma hyorhinis P37 protein using monoclonal antibodies against baculovirus-expressed P37 protein

**DOI:** 10.1186/s12866-019-1614-4

**Published:** 2019-11-05

**Authors:** Hongzhen Zhu, Yanwu Wei, Liping Huang, Dan Liu, Yongxing Xie, Deli Xia, Haiqiao Bian, Li Feng, Changming Liu

**Affiliations:** 1 Division of Swine Digestive System Infectious Diseases, State Key Laboratory of Veterinary Biotechnology, Harbin Veterinary Research Institute, Chinese Academy of Agricultural Sciences, No. 678 Ha-ping Street, Xiang-fang region, Harbin, 150069 China; 2 College of Veterinary Medicine, Ji Lin University, Changchun, 130062 China

**Keywords:** *Mycoplasma hyorhinis*, P37 protein, Monoclonal antibody, Antigenic epitope

## Abstract

**Background:**

*Mycoplasma hyorhinis* (Mhr) is the etiologic agent of lameness and polyserositis in swine. P37 is a membrane protein of Mhr that may be an important immunogen and is a potential target for diagnostic development. However, there is little information concerning Mhr P37 protein epitopes. A precise analysis of the P37 protein epitopes should extend our understanding of the antigenic composition of the P37 protein and the humoral immune responses to Mhr infection. Investigating the epitopes of Mhr P37 will help to establish a detection method for Mhr in tissue and provide an effective tool for detecting Mhr infection.

**Results:**

Western blot and indirect immunofluorescence assays (IFA) confirmed that the expressed P37 protein was recognized by Mhr-positive porcine and mouse sera. Furthermore, the P37 protein was purified using affinity chromatography and used to immunize mice for hybridoma cell fusion. Four monoclonal antibodies (mAbs) found to be positive for Mhr were detected in infected lung tissue. A panel of truncated P37 proteins was used to identify the minimal B cell linear epitopes of the protein based on these mAbs. The core epitope was determined to be ^206^KIKKAWNDKDWNTFRNF^222^_._

**Conclusions:**

In this study, we identified 17 critical amino acids that determine the epitope of the P37 protein of Mhr. This study identified mAbs that could provide useful tools for investigating the Mhr P37 antigenic core epitope (amino acids 206–222) and detecting Mhr-specific antigens in infected tissue.

## Background

*Mycoplasma hyorhinis* (Mhr) was first isolated in 1953 and found to lack a cell wall [1]. It is a commensal microorganism that inhabits the upper respiratory tract of swine [2]. Mhr infections in pigs can cause lameness and polyserositis, and severe infections can cause pneumonia [3, 4]. Systemic infection caused by Mhr is found on pig farms worldwide and is characterized by high morbidity and low mortality rates [5, 6]. At present, Mhr infection detection mainly depends on pathogen isolation and culture and polymerase chain reaction (PCR) methods; and there is no commercially available kit for serological detection, as Mhr is a commensal in the respiratory tract and tonsils of pigs, presence of antibodies does not indicate M. hyorhinis as an etiologic agent of clinical signs [2, 7]. Although Mhr is easily isolated from porcine alveolar lavage fluid and nasal swabs, the process of isolation and identification of Mhr is often time consuming [8]. Mhr has been proven to be a zoonotic pathogen and identified in co-infection with PRRSV or PCV2 in the porcine respiratory system [9–12]. In general, the treatment of Mhr infection is mainly through the use of antibiotics [5].

Members of the variable lipoprotein family of Mhr have been shown to perform a variety of adherence functions during infection and interactions with the host, which presumably facilitates chronic infections [13, 14]. P37 is an important membrane protein of Mhr and is part of the periplasmic binding protein-dependent transport system [15, 16]. P37 may play a role in tumor invasion, and detection of antibodies against P37 in human serum may help diagnose cancer [17, 18].

Previously, the P37 protein was used as a coating antigen to measure the immunoglobulin G (IgG) responses in swine vaccinated with an inactivated Mhr vaccine [19]. However, it was unclear whether P37 protein could be used as an accurate indicator to identify naturally infected pigs in lung tissues, the role of P37 in the process of infection, or the precise epitope of P37.

In this study, we used monoclonal antibodies (mAbs) prepared in mice based on P37 protein expressed using the baculovirus expression system to detect the distribution of Mhr in infected tissues by immunohistochemistry and identify the core epitope of P37 protein using the truncated protein method.

## Results

### Identification of recombinant plasmid and shuttle plasmid

**Fig. 1:**
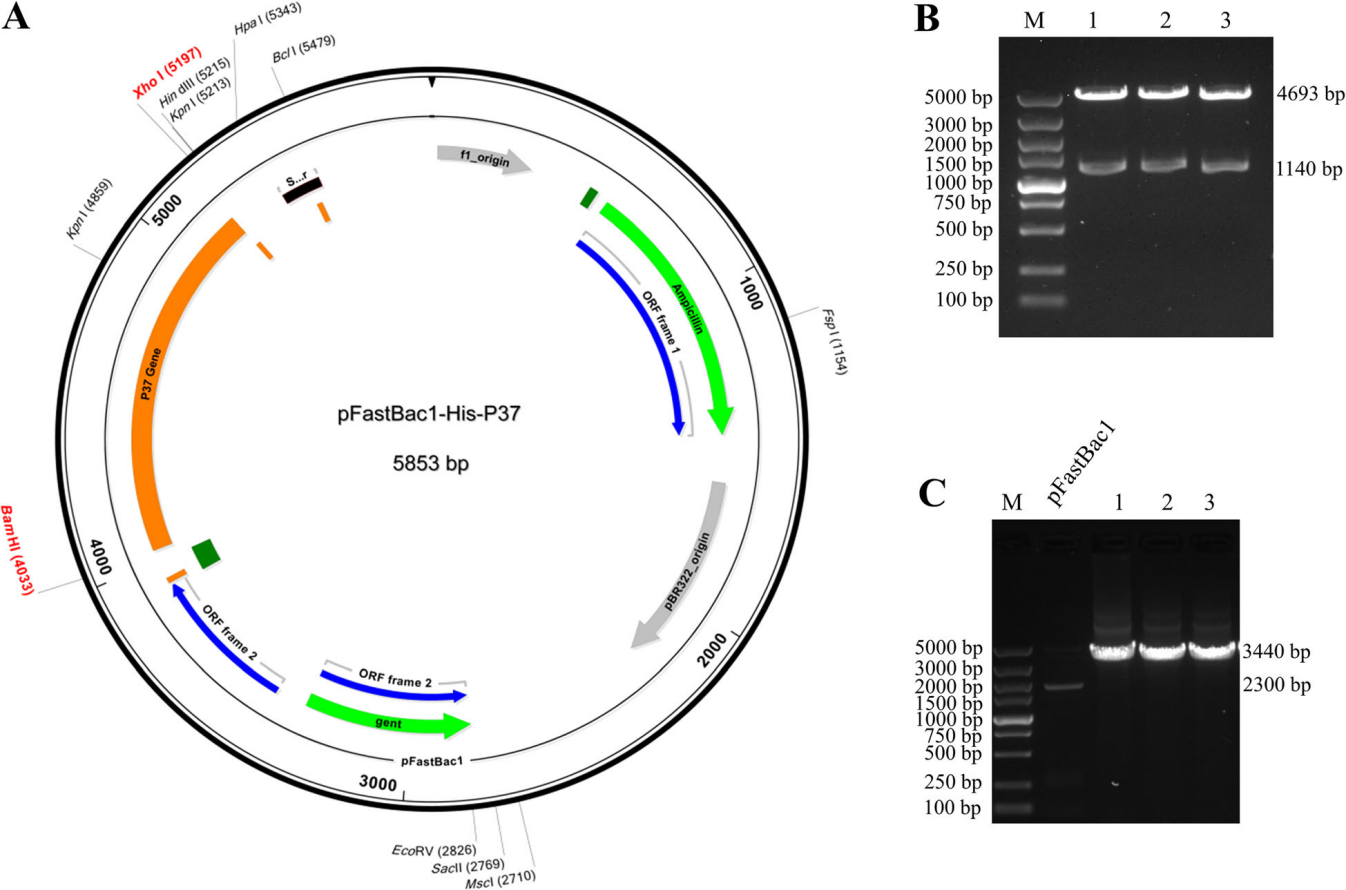
Identification of recombinant plasmid and shuttle plasmid. **a** The restriction map and primer-binding sites. The P37 gene (1140 bp) was subsequently cloned into the expression vector pFastBac™1 via two restriction sites (*Bam*H I and *Xho* I). **b** Double enzyme digestion (*Bam*H I and *Xho* I) revealed specific bands at 4693 bp and 1140 bp. Lane M indicates the DNA molecular quality standard. Lanes 1, 2, and 3 represent plasmids extracted after single-colony expansion of randomly selected white colonies based on the Bac-to-Bac® Baculovirus Expression System instruction Version D (Invitrogen, Carlsbad, CA, USA). **c** The pFastBac™1-His-P37 shuttle plasmid was identified using M13 primers, and a specific band appeared at 3440 bp, whereas pFastBac1 showed a specific band at 2300 bp. Lane M indicates the DNA molecular quality standard. Lane pFastBac1 indicates the pFastBac™1 plasmid negative control. Lanes 1, 2, and 3 represent plasmids extracted after single-colony expansion of randomly selected white colonies as described above

The recombinant plasmid pFastBac™1-His-P37 was identified by dual-restriction endonuclease digestion with *Bam*H I and *Xho* I, and the 4693 bp vector fragment and the 1140 bp target gene fragment were visualized by 1% agarose gel electrophoresis (Fig. 1). pFastBac™1-His-P37 was specifically amplified using M13 primers, and a 3440 bp band was obtained on a 1% agarose gel. The negative control pFastBac™1 was observed as a 2300 bp fragment (Fig. 1).

### Detection and purification of recombinant protein

**Fig. 2:**
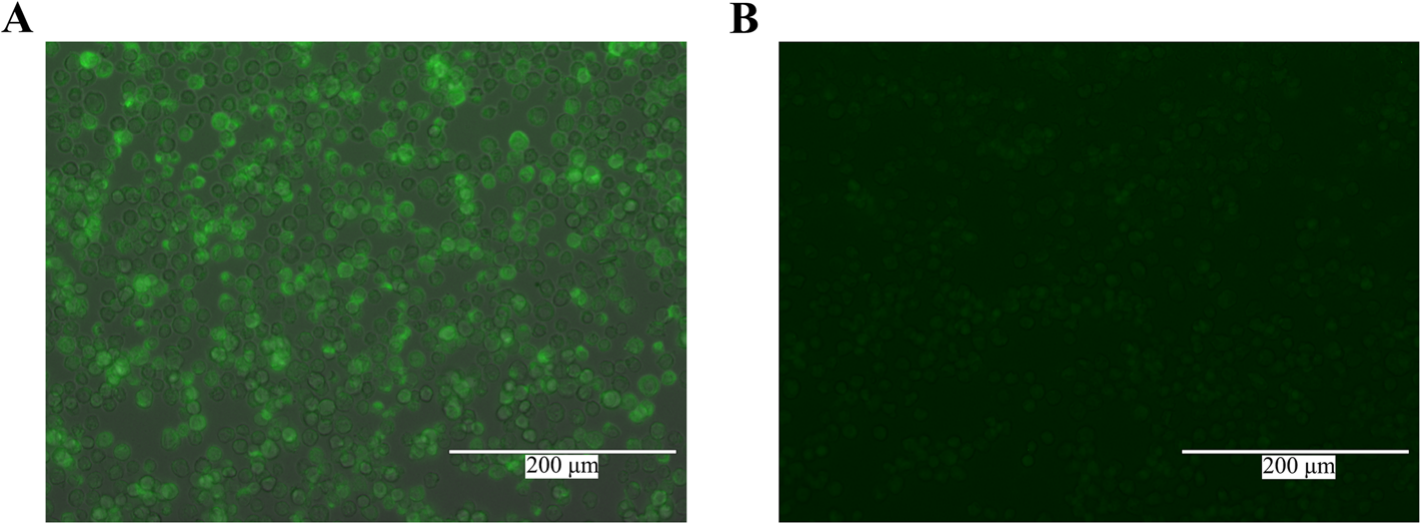
Detection of recombinant P37 protein by indirect immunofluorescence assay (IFA). **a** Detection of Sf21 cells infected with pFastBac™1-His-P37 by IFA. Infected insect cells showed strong fluorescence. **b** Detection of uninfected Sf21 cells by IFA. No fluorescence was detected from negative cells

**Fig. 3:**
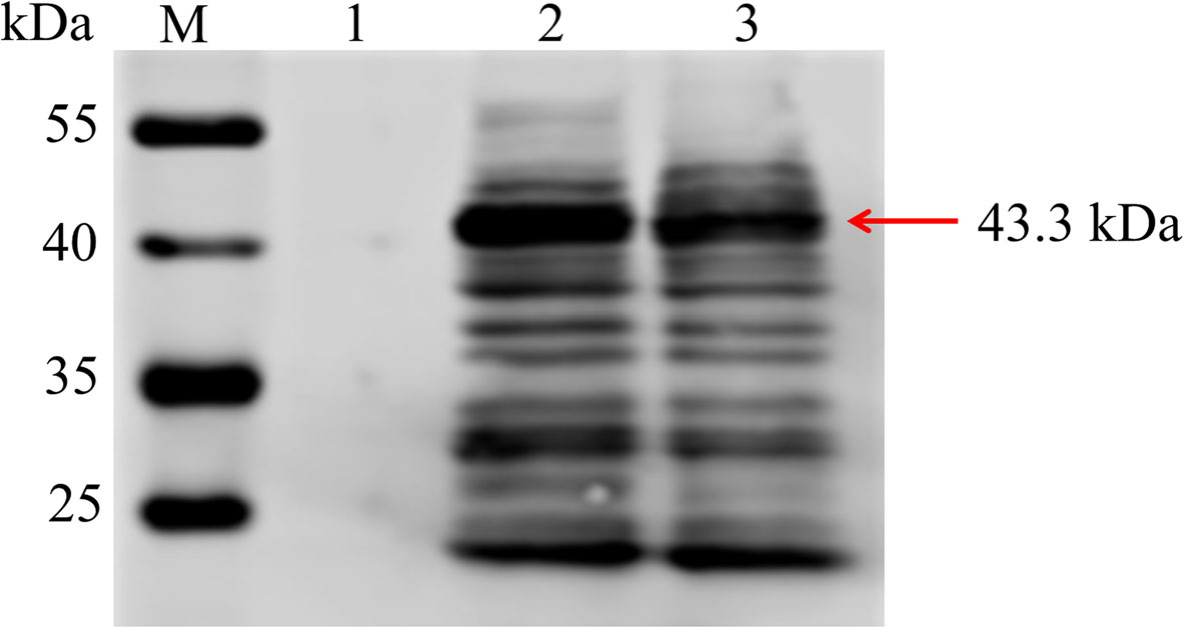
P37 protein identified by Western blot analysis. Lane M, PageRuler™ Prestained Protein Ladder (Thermo, Vilnius, Lithuania); lane 1, cell lysate from normal Sf21 cells; lane 2, recombinant P37 protein expressed in the baculovirus expression system; lane 3, recombinant P37 protein purified from the baculovirus expression system

Using fluorescence microscopy, strong fluorescence was observed in insect cells infected with pFastBac™1-His-P37, whereas no fluorescence was observed in uninfected cells (Fig. 2), indicating that the P37 protein was successfully expressed in insect cells. Western blot analysis showed that the protein reacted with the prepared anti-Mhr mouse positive serum, and a specific reaction band appeared at approximately 43.3 kDa (Fig. 3).

### Characterization of P37 protein-specific mAbs

**Fig. 4:**
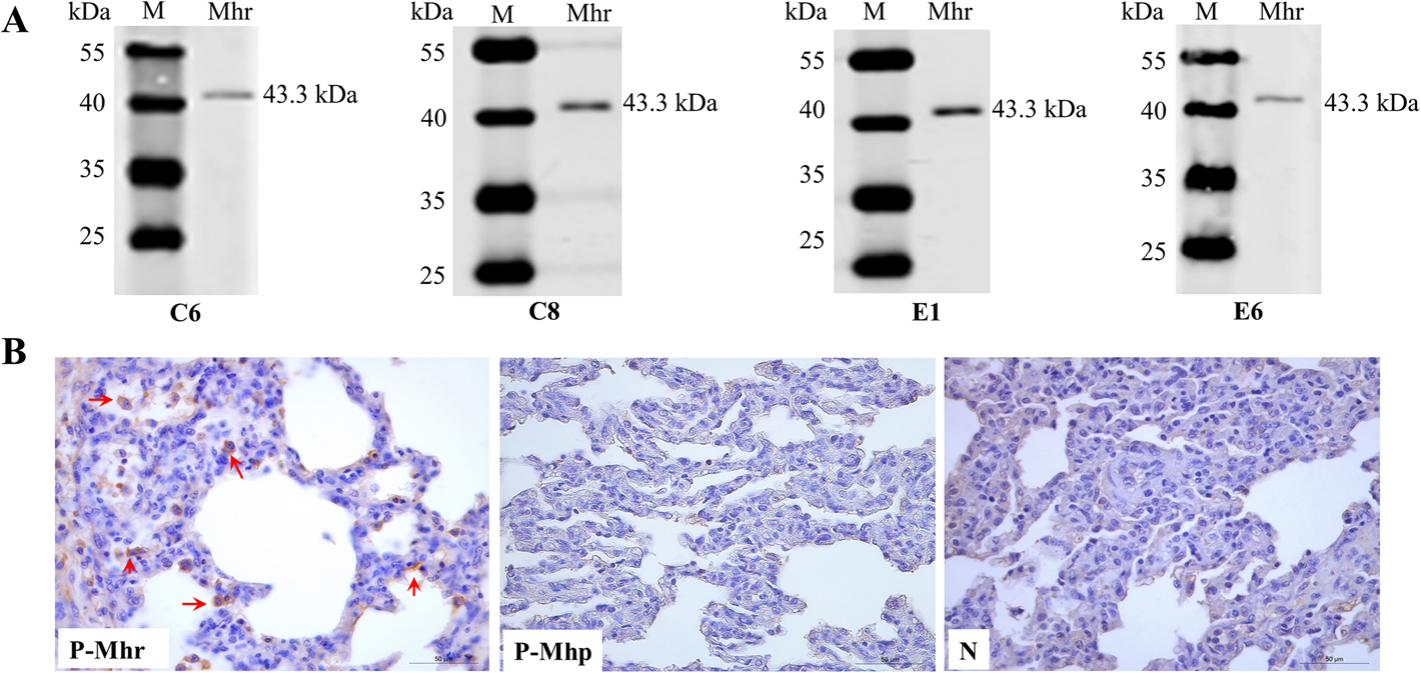
Western blot analysis of four mAbs to Mhr. **a** Western blot analysis revealed four mAbs (C6, C8, E1, and E6). A specific Mhr band was detected at 43.3 kDa, indicating that the P37 protein expressed by the eukaryotic expression system had good immunogenicity. Lane M, PageRuler™ Prestained Protein Ladder; lane Mhr, Mhr lysate. **b** Immunohistochemical analysis of Mhr-specific antigens in infected lung tissue using prepared mAbs. Positive signals (red arrows) were detected for the Mhr-specific antigen in infected lung tissue (P-Mhr) but were not detected in Mhp positive lung tissue (P-Mhp) and uninfected lung tissue (N)

The four positive mAbs were designated C6, C8, E1, and E6. Subtype identification results showed that the heavy chain subtype of all four mAbs was IgG1, and the light chain subtype of all mAbs was kappa (Table 1). Reactivity analysis of mAbs to Mhr showed that the Mhr strain reacted specifically with the four mAbs, and a specific reaction band appeared at a position of 43.3 kDa (Fig. 4). Immunohistochemical detection showed that Mhr-specific antigen was present in the lungs of confirmed Mhr-positive cases, and Mhr-specific antigen mainly localized to epithelial cells of lung tissues, with no Mhr-specific antigen and Mhp-specific antigen observed in uninfected lung tissue and Mhp positive lung tissue (Fig. 4).

**Table 1:** Identification of subclasses of mAbs in hybridoma cell supernatants

	Hybridoma			
	C6	C8	E1	E6
Ig subclass	IgG1	IgG1	IgG1	IgG1
Light chain type	Κ	Κ	Κ	Κ

### Identification of B cell linear epitopes of P37 using P37-specific mAbs

**Fig. 5:**
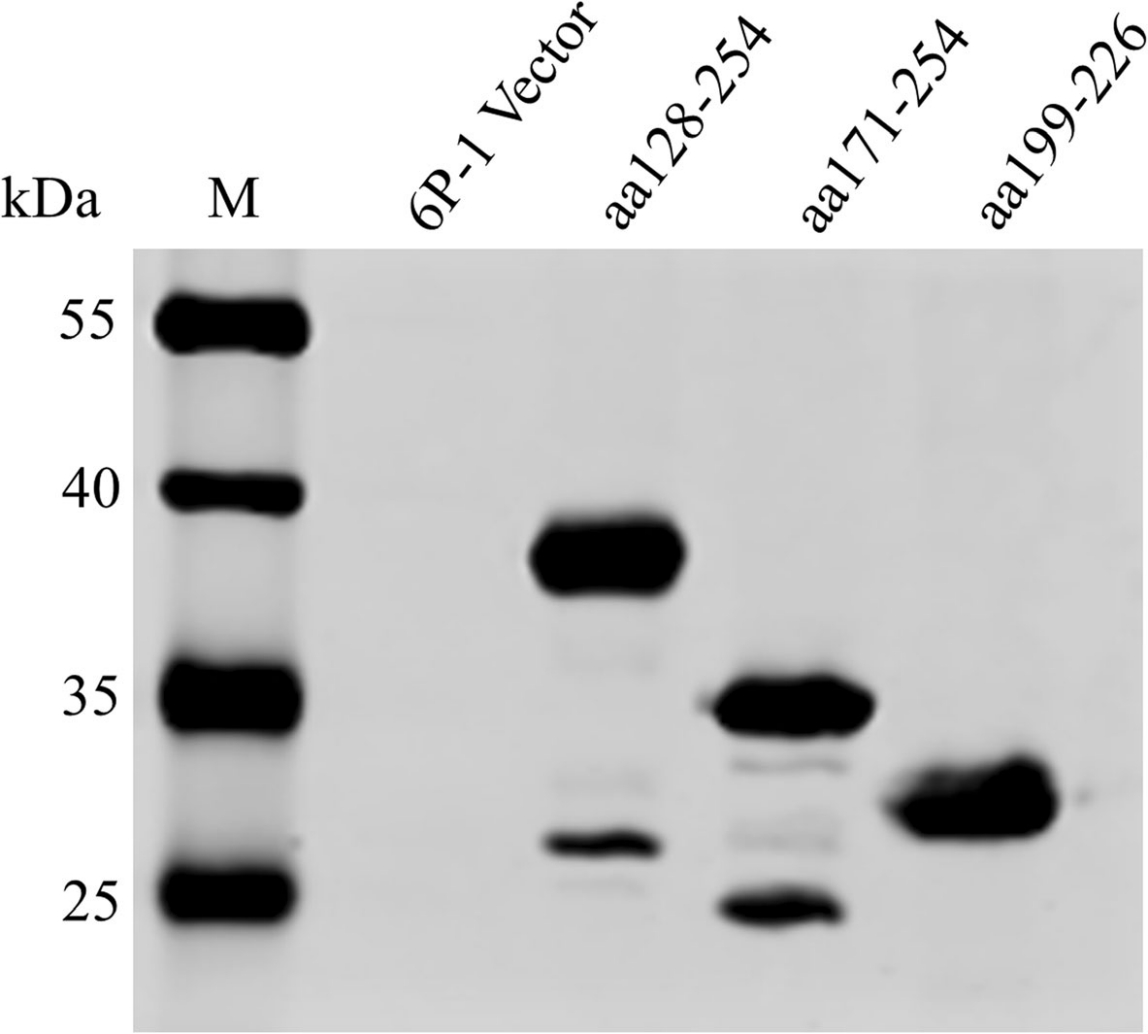
Western blot analysis of mAbs that reacted specifically with the P37 protein fragment. Western blot analysis showed that mAbs reacted specifically with aa128–254, aa171–254, and aa199–226, and the results were consistent for the four mAbs. Lane M, marker; lane 6P-1, vector lysate after IPTG induction

To determine the epitopes of the four generated mAbs, a series of overlapping peptides were analyzed by Western blot analysis. The results showed the presence of specific bands at amino acids (aa) 128–254, aa171–254, and aa199–226 (Fig. 5). Because the four mAbs were identical in reactivity, only one result is shown here.

**Fig. 6:**
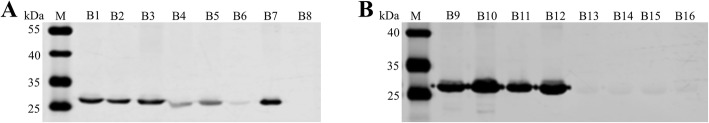
Western blot analysis verification of key amino acids. In order to identify key amino acids, peptides were deleted one by one. B1–B8 indicates deleted peptides aa209–206, respectively. B9–B16 indicates deleted peptides aa226–219, respectively. **a** No specific band was detected when ^206^Lys was deleted. **b** No specific band was detected when ^222^Phe was deleted

To confirm the epitopes of the mAbs, Western blot analysis was performed after mutating each aa one by one. No specific bands were detected after deletion of aa ^206^Lys (Fig. 6a) and aa ^222^Phe (Fig. 6b). This indicates that aa ^206^Lys and aa ^222^Phe are key amino acids of the core epitope of the P37 protein.

### Analysis of P37 protein from different Mhr strains

**Fig. 7:**
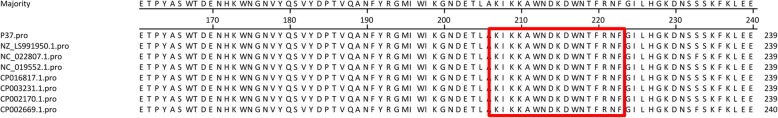
Alignment of sequences with Mhr P37 epitopes. A total of seven Mhr strains were analyzed. The sequence motif recognized by mAbs is shown in the red box

Analysis of the p37 sequence of seven Mhr strains demonstrated that the epitope ^206^KIKKAWNDKDWNTFRNF^222^ was highly conserved (100% aa identity, Fig. 7).

### Homology modeling of the P37 protein epitope

**Fig. 8:**
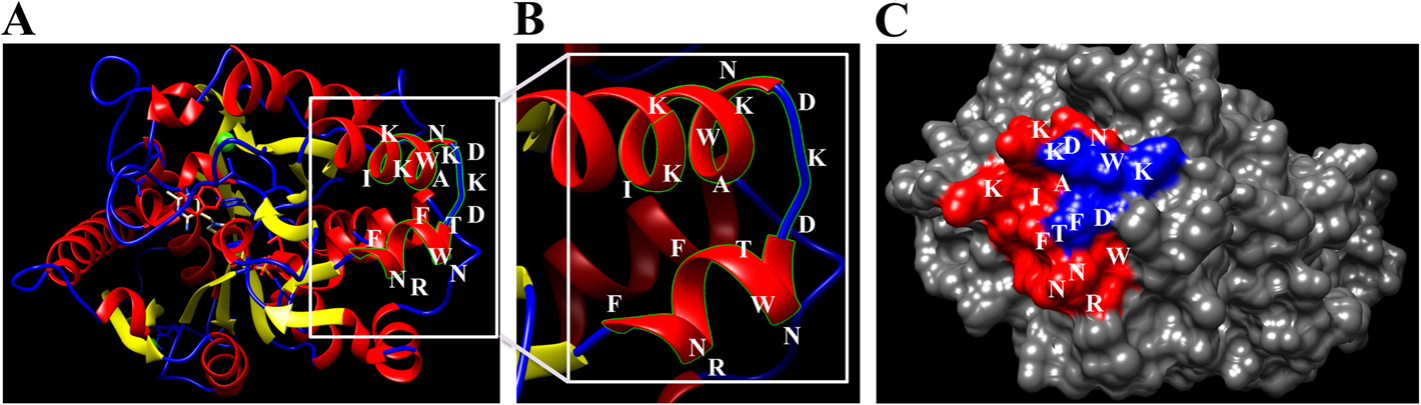
Structural analysis of the P37 protein. Structural analysis and identification of B cell epitopes in the predicted three-dimensional structure of the P37 protein. Red areas represent α-helix regions, blue areas represent nonregular coil regions, and yellow areas represent strand regions. **a** The location of epitope ^206^KIKKAWNDKDWNTFRNF^222^ is shown in a gray box. **b** Areas where antigen reacted to antibodies. **c** The epitope was located on the surface of the P37 protein

The spatial structure of the P37 protein from aa1–379 was predicted using three-dimensional homology modeling. Model analysis showed that the overall shape of P37 is an irregular prolate ellipsoid, and the core epitope domain consists of two α-helices and a nonregular coil (Fig. 8a and b). The epitope region where the antigen-antibody reaction was detected was located on the surface of the P37 protein (Fig. 8c).

## Discussion

A previous study suggested that Mhr was present in serum samples from pigs at fattening farms [20]. Although most pigs infected with Mhr do not show significant clinical symptoms, typical lesions found in infected 1-day to 1-year-old pigs include serum fibrotic pleurisy, otitis media, pericarditis and peritonitis, which may cause fibrous adhesions during the chronic phase [21–23]. Hence, accurate diagnosis is critical to establish effective treatment measures in affected herds.

P37 is thought to function in adherence to host cells and has been used as an antigen to monitor the production of antibodies in animals immunized with inactivated Mhr vaccine [17, 19, 24]. In this study, we focused on the conserved P37 protein of Mhr as a potential target for diagnostic development. We used mAbs prepared in mice based on P37 protein expressed using the baculovirus expression system to establish an immunohistochemical method, which can be used to detect Mhr-specific antigen in naturally infected tissues. A precise analysis of P37 protein epitopes should extend our understanding of the antigenic composition of the P37 protein.

In this study, four P37-specific mAbs were produced using His-fused P37 expressed in sf21 cells as the immunogen. A series of GST-fused partial regions of P37 expressed in *Escherichia coli* were used to identify linear B cell epitopes in the P37 protein. Western blotting analysis showed that the four antibodies identified the same epitope, ^206^KIKKAWNDKDWNTFRNF^222^. We speculated that the P37 protein expressed in sf21 cells was modified compared to that expressed in *E. coli*, which is consistent with previously described results [18]. P37 protein expressed in sf21 cells has post-translational modifications compared to P37 protein expressed in *E. coli*. However, the mechanism of P37 protein modification in sf21 cells is unknown and requires further study.

Based on alignment of P37 amino acids, sequence analysis of different Mhr strains showed that the ^206^KIKKAWNDKDWNTFRNF^222^ epitope was completely conserved in all Mhr strains. Although antigenic epitopes are usually contained within aa6–10, interestingly this epitope region was in aa17 [25, 26]. Structural predictions by 3D homology modeling revealed that the epitope is exposed on the surface of the P37 protein and includes a non-regular coil. The fact that the functional part of the enzyme was often in the conformational region of the non-regular coil indicates that the establishment of an epitope-based diagnostic method could potentially exploit antigens for Mhr detection and avoid cross-reactions with *Mycoplasma hyopneumoniae* (Mhp) [27].

Immunohistochemical analysis showed that mAbs against the P37 protein can be applied to detect Mhr antigen in the lung tissue of Mhr-infected animals. Mhr mainly infects epithelial cells in the lungs, which is consistent with previously described results [28]. Therefore, we believe that mAbs against P37 protein expressed in sf21 cells could be used to detect Mhr infection in pigs.

## Conclusions

This study identified the core epitope of the P37 protein as ^206^KIKKAWNDKDWNTFRNF^222^. Homology modeling analysis showed that this epitope consists of two α-helices and a non-regular coil. Immunohistochemistry results indicated that the epitope of the P37 protein may be involved in the production of host antibodies.

## Methods

### Pathogens, plasmids, cells, and anti-Mhr serum

The previously described Mhr-DL strain (CGMCC No. 11092) was used as the coating antigen for screening and Western blot analysis of mAbs [29]. Anti-Mhr mouse serum was prepared using the Mhr-DL strain as an immunogen. The baculovirus transfer vector pFastBac™1 (Invitrogen, Carlsbad, CA, USA) was used to construct the pFastBac™1-His-p37 plasmid, which expressed the complete P37 protein. The prokaryotic expression vector pGEX-6P-1 (GE Healthcare, Uppsala, Sweden) was used to express the truncated P37 protein. The insect cell line *Spodoptera frugiperda* (sf21) (Invitrogen, Carlsbad, CA, USA) was cultured in Grace’s Insect Medium (Invitrogen) at 27 °C to propagate recombinant baculovirus. The SP2/0 myeloma cell line (ATCC® CRL-1581™) was cultured in Dulbecco’s modified Eagle’s medium (DMEM) (Invitrogen, Grand Island, NY, USA) with 10% inactivated fetal bovine serum (FBS, Thermo Fisher Scientific, North Shore City, New Zealand) in a humidified incubator with 5% CO_2_ at 37 °C and was then fused with mouse spleen cells.

The mouse anti-Mhr serum was prepared as follows: M. hyrohinis (1 × 10^10.0^ CCU/mL) mixed to a concentration of 15% (v/v) with Seppic Montanide ISA15 VG (www.seppic.com), and then one 6-week-old female BALB/c mice were immunized with 500 μL. This immunization was repeated 3 weeks later and after further 2 weeks, mice were euthanized and collected blood, and then centrifugation 8000 rpm/min for 10 min at 4 °C.

### Acquisition of the p37 gene and generation of recombinant baculovirus

The p37 gene fragment was amplified by PCR with forward (5′-CGGGATCCATGCTGAAGAAGCTGAAG-3′) and reverse (5′-CCGCTCGAGTTACTTGATGGCCTTCTC-3′) primers designed using Premier 5.0 software (PREMIER Biosoft, Palo Alto, CA, USA) based on the reference sequence (GenBank Accession No. X14140.1). The signal peptide sequence was removed from the p37 gene coding region and the p37 gene was optimized and synthesized by BGI Co. (Beijing, China). For protein purification, a 6× His-tag was fused to the NH_2_-terminal end of the p37 gene. The gene sequence was optimized to obtain the highest possible level of expression, and the target gene (1140 bp) was subsequently cloned into the expression vector pFastBac™1 via two restriction sites (*Bam*H I and *Xho* I) (Fig. 1).

The linearized baculovirus DNA was transformed into competent DH10Bac (Invitrogen) according to the manufacturer’s instructions. Identification of recombinant baculovirus was carried out using universal M13 primers (forward primer: 5′-GTTTTCCCAGTCACGAC-3′, reverse primer: 5′-CAGGAAACAGCTATGAC-3′). pFastBac™1-His-P37 was transfected into logarithmic phase Sf21 insect cells according to the Bac-to-Bac® Baculovirus Expression System instructions (Invitrogen). When the cells exhibited obvious cytopathic effects, the cell supernatant was collected as the first generation recombinant baculovirus and designated P1 baculovirus. P1 baculovirus was then used to infect cells by performing a viral plaque assay to generate a high viral titer of P2 baculovirus. Prepared P2 baculovirus was used for expression studies.

### Expression and purification of P37 protein

For P37 protein expression, three generations of high titer seed virus stocks were prepared by infecting Sf21 cells at a multiplicity of infection (MOI) of 0.1 plaque forming units (PFUs). Cells in 24-well plates were infected with pFastBac™1-His-P37 baculovirus, and uninfected Sf21 cells were used as mock control. The cell culture medium was aspirated after 60 h and fixed with 10% paraformaldehyde (500 μL per well) at room temperature for 15 min. The Sf21 cells were then fixed with 0.2% TritonX-100 for 10 min, washed three times with phosphate buffered saline (PBS), and incubated with mouse anti-Mhr serum (Anti-Mhr serum was prepared and stored by our laboratory, and diluted 1:500 in PBS) at 37 °C for 1 h. The cells were then washed three times with PBS, incubated with DyLight 488 AffiniPure Goat Anti-Mouse IgG (H + L) (diluted 1:500 in PBS; Pierce, Rockford, IL, USA) at 37 °C for 1 h, and washed three times with PBS. The fluorescent signal was visualized with an EVOS inverted fluorescence microscope (Life Technologies, Carlsbad, CA, USA).

Sf21 cells infected with 5 MOI pFastBac™1-His-P37 virus were harvested 72 h post infection. Cells were suspended in PBS (1% of the original volume), lysed by ultrasonic lysis for 30 min (pulse on 3 s, pulse off 5 s, 130 watts), centrifuged at 9000 rpm for 30 min at 4 °C to remove the precipitate, and purified using Ni-NTA affinity chromatography (Genscript, Nanjing, China). The purified sample was mixed with 5× loading buffer at a ratio of 4:1 and boiled for 10 min. The expressed protein was separated by 12% sodium dodecyl sulfate-polyacrylamide gel electrophoresis (SDS-PAGE) and transferred to a nitrocellulose membrane. The membrane was blocked with 5% skim milk powder in PBS solution overnight at 4 °C, incubated with anti-Mhr mouse serum (1:100) at room temperature for 1 h, and washed three times with PBS containing 0.5% Tween 20 (PBST). After incubation for 1 h at room temperature with DyLight™ 680-Labeled Antibody To Mouse IgG (H + L) (1:10000) (KPL, Gaithersburg, MD, USA), washed three times with PBST, and scanned using an Odyssey infrared imaging system (Licor Odyssey, Lincoln, NE, USA).

### Preparation of mAbs against P37 protein

Four 6-week-old female BALB/c mice were purchased from the Laboratory Animal Center of Harbin Veterinary Research Institute, CAAS (Harbin, China). The mice were immunized with 80 μg of the purified P37 protein. Protein emulsification and immunization were performed as previously described [30]. P37 protein emulsified with complete Freund’s adjuvant (Sigma-Aldrich) was injected subcutaneously. After an interval of 3 weeks, the mice were boosted with P37 protein emulsified in incomplete Freund’s adjuvant (Sigma-Aldrich). After a 2-week interval, mice were intraperitoneally administered 80 μg of P37 protein without adjuvant. On the 3 d of intraperitoneal immunization, the mice were euthanized by cervical dislocation (disconnecting the spinal cord from the brain, causing the experimental animals to die without pain), and their splenocytes were fused with SP2/0 cells as previously described [31]. The fused cells were mixed with DMEM medium containing hypoxanthine-aminopurine-thymidine (HAT) (Sigma-Aldrich, New York, NY, USA) and 20% FBS. Cells were cultured together and the media were replaced with selection medium containing hypoxanthine-thymidine (HT) (Sigma-Aldrich) and 10% FBS after 5 d [25]. Hybridoma supernatants were collected after 7 d and screened for the presence of Mhr-specific antibodies by indirect enzyme-linked immunosorbent assay (ELISA) [28]. The purified P37 protein was used as the coating antigen at a concentration of 5 ng/μL as previously described [28]. Positive hybridoma cells were cloned three times by limiting dilution and stored in liquid nitrogen.

### Characterization of mAbs against P37 protein

The subtypes of mAbs produced were determined using the SBA Clonotyping System-HRP kit (Southern Biotech, Birmingham, AL, USA) according to the manufacturer’s instructions. The hybridoma supernatant was added as a primary antibody and horseradish peroxidase (HRP)-conjugated goat anti-mouse antibody was used as the secondary antibody. Color development and screening were performed as described above. The reactivity of mAbs to Mhr was determined by Western blot analysis as described above and Mhr-specific antigen in the lung tissue of Mhr-infected pigs was detected using immunohistochemical assays. The proteolytic antigen retrieval step of the immunohistochemical assay was modified to include proteinase K (Merck Life Science Co. Ltd., Shanghai, China). Uninfected lung tissue (by PCR detection) was used as the negative control and the cross reactivity of mAbs to Mhp was tested by immunohistochemistry using Mhp positive tissues.

### Preliminary identification of P37 protein B cell line epitopes using P37-specific mAbs

**Fig. 9:**
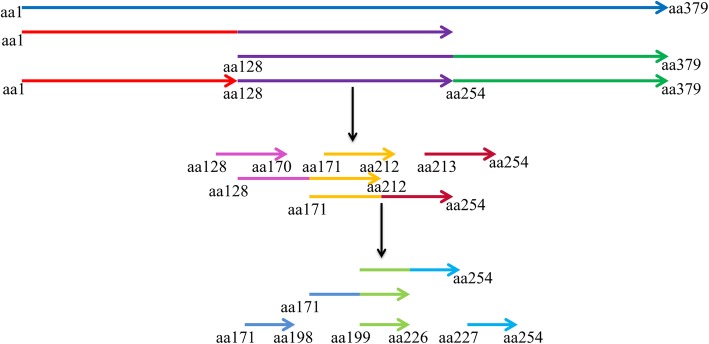
P37 protein fragment truncation protocol. Schematic representation of the protocol for P37 protein epitope identification by fragment truncation. The black arrow represents regions (aa128–254, aa171–254, and aa199–226) that reacted with the four prepared mAbs

To identify the epitopes of the mAbs produced against the P37 protein, a series of nucleotide sequences encoding aa regions of P37 were cloned into the *Bam*H I and *Xho* I sites of pGEX-6P-1 (Fig. 9). After sequencing, *E. coli* BL-21 cells were transformed with the recombinant plasmids, which were induced by the addition of 1 mM isopropyl β-D-1-thiogalactopyranoside (IPTG) and incubated at 37 °C with shaking for 6 h. Cultures were harvested and lysed, and lysates were analyzed by SDS-PAGE and Western blot. Lysate from induced pGEX-6p-1 *E. coli* BL-21 cells was used as the negative control. The prepared mAbs against the P37 protein were used as the primary antibody, and DyLight™ 680-Labeled Antibody to Mouse IgG (H + L) (1:10,000) was used as the secondary antibody. The plate was incubated for 1 h at room temperature in the dark, washed three times with PBST, and analyzed using the Odyssey infrared imaging system (Licor Odyssey).

### Precise localization of the P37 protein B cell epitope

To define the minimal linear epitope of the P37 protein, aa199–226 were deleted one by one (listed in Table 2) and their corresponding peptides were assessed by Western blot as described above. The immunoreactivity of the core epitope to corresponding mAbs was then analyzed.

**Table 2:** Peptides used in this study and their corresponding amino acid sequences

Peptide	Peptide sequence
B1 (aa200–226)	NDETLAKIKKAWNDKDWNTFRNFGILH
B2 (aa201–226)	DETLAKIKKAWNDKDWNTFRNFGILH
B3 (aa202–226)	ETLAKIKKAWNDKDWNTFRNFGILH
B4 (aa203–226)	TLAKIKKAWNDKDWNTFRNFGILH
B5 (aa204–226)	LAKIKKAWNDKDWNTFRNFGILH
B6 (aa205–226)	AKIKKAWNDKDWNTFRNFGILH
B7 (aa206–226)	KIKKAWNDKDWNTFRNFGILH
B8 (aa207–226)	IKKAWNDKDWNTFRNFGILH
B9 (aa199–225)	GNDETLAKIKKAWNDKDWNTFRNFGIL
B10 (aa199–224)	GNDETLAKIKKAWNDKDWNTFRNFGI
B11 (aa199–223)	GNDETLAKIKKAWNDKDWNTFRNFG
B12 (aa199–222)	GNDETLAKIKKAWNDKDWNTFRNF
B13 (aa199–221)	GNDETLAKIKKAWNDKDWNTFRN
B14 (aa199–220)	GNDETLAKIKKAWNDKDWNTFR
B15 (aa199–219)	GNDETLAKIKKAWNDKDWNTF
B16 (aa199–218)	GNDETLAKIKKAWNDKDWNT

### Multiple alignment of P37 amino acid sequences

Multiple alignments of aa sequences of the P37 protein of seven Mhr isolates (GenBank accession Nos. CP002170.1, CP002669.1, CP003231.1, CP016817.1, NC_019552.1, NC_022807.1, and NZ_LS991950.1) were performed using the Clustal W method within DNASTAR software version 7.0 (https://www.dnastar.com/software/).

### Homology modeling of P37 epitopes

Homology modeling of aa1–379 of the P37 protein was performed using SWISS-MODEL (https://www.swissmodel.expasy.org/interactive). The spatial locations of the identified P37 protein epitopes were determined by mapping the epitopes to a three-dimensional model of the P37 protein using Chimera 1.11.2 software (https://www.cgl.ucsf.edu/chimera/).

## Data Availability

The datasets used and/or analyzed during the current study are available from the corresponding author on reasonable request. All data generated or analyzed during this study are included in this published article.

## Abbreviations

aa: amino acid
CCU: color change unit
DMEM: Dulbecco’s modified Eagle’s medium
ELISA: enzyme-linked immunosorbent assay
FBS: fetal bovine serum
HRP: horseradish peroxidase
HT: hypoxanthine-thymidine
IFA: indirect immunofluorescence assays
IgG: immunoglobulin G
IPTG: isopropyl β-D-1-thiogalactopyranoside
kDa: kilodalton
Lys: lysine
mAbs: monoclonal antibodies
Mhr: *Mycoplasma hyorhinis*
MOI: multiplicity of infection
PBS: phosphate buffered saline
PBST: PBS containing Tween 20; HAT hypoxanthine-aminopurine-thymidine
PCR: polymerase chain reaction
PFUs: plaque forming units
Phe: phenylalanine
SDS-PAGE: sodium dodecyl sulfate-polyacrylamide gel electrophoresis
sf21: *Spodoptera frugiperda*
Κ: kappa
